# Flow-Based Systems for Rapid and High-Precision Enzyme Kinetics Studies

**DOI:** 10.1155/2012/450716

**Published:** 2012-04-22

**Authors:** Supaporn Kradtap Hartwell, Kate Grudpan

**Affiliations:** ^1^Department of Chemistry, Xavier University, 3800 Victory Parkway, Cincinnati, OH 45207, USA; ^2^Department of Chemistry, Faculty of Science, Chiang Mai University, Chiang Mai 50200, Thailand; ^3^Center of Excellence in Innovation for Analytical Science and Technology, Chiang Mai University, Chiang Mai 50200, Thailand

## Abstract

Enzyme kinetics studies normally focus on the initial rate of enzymatic reaction. However, the manual operation of steps of the conventional enzyme kinetics method has some drawbacks. Errors can result from the imprecise time control and time necessary for manual changing the reaction cuvettes into and out of the detector. By using the automatic flow-based analytical systems, enzyme kinetics studies can be carried out at real-time initial rate avoiding the potential errors inherent in manual operation. Flow-based systems have been developed to provide rapid, low-volume, and high-precision analyses that effectively replace the many tedious and high volume requirements of conventional wet chemistry analyses. This article presents various arrangements of flow-based techniques and their potential use in future enzyme kinetics applications.

## 1. Introduction

Enzymes are generally referred to as globular proteins that are comprised of a few tenths to thousandths of amino acid residues, where only a small part (a few amino acids acting as binding sites) of the enzyme molecule is actually involved in catalysis [1, 2]. However, some studies showed that enzymatic ability may not be restricted to proteins only, and a simple amino acid, so called proline, may be considered as an enzyme due to its ability to catalyze certain chemical reactions [3]. Nevertheless, substances that are categorized as enzymes have distinctive features in common, that is, specificity toward a certain molecule (usually through physical shape, size and chemical properties at the binding sites), ability to speed up the reaction, and possession of a turnover feature where the same enzyme molecule keeps its reactivity after each catalytic reaction and can react again and again with more incoming substrate molecules [4].

Enzyme studies are important for numerous applications. Study of enzyme kinetics is always an important part of the discovery of enzyme characteristics. Suitable substrate that enhances the efficiency of enzyme can be selected based on enzyme kinetics parameters. Similarly, enzyme inhibitor which may be avoided or may be required depending on objectives of use can also be revealed from enzyme kinetic studies [4–6].

Enzymes act as catalysts, thus, they are not consumed. Therefore, activity of an enzyme is usually detected from the rate of change in concentration of either the products being produced or the substrate being used. At the beginning of enzyme-substrate reactions, the concentration of a product increases rapidly and linearly. As the reaction progresses further, the rate slows down due to accumulation of product and lessening of substrate. Even though steady state enzyme kinetic studies may be performed using a nonlinear relationship of enzyme activity and product concentration (by taking measurement of changes in product or substrate concentrations after completion of the reaction), the linear initial rate (presteady state) is preferred for best accuracy. Presteady state kinetics study also offers benefits in terms of rapidity, simplicity of linear relationship between enzyme activity and concentration of product, and minimized consumption of expensive or rare enzymes. However, in many cases, initial rate study is difficult due to the speed of some enzymes where the initial rate may last only a few seconds [6, 7]. Typically, in order to estimate kinetic parameters such as Michaelis-Menten constant (*K*_*m*_) and maximum velocity (*V*_max⁡_), solutions with fixed concentration of enzyme are mixed with various concentrations of substrate in different cuvettes and then observation is made of the spectrophotometric changes caused by the enzyme-substrate reaction at the beginning of the reaction. With manual operation, this poses a difficulty in precise measurement of product or substrate concentration at initial stage of reaction. Analytical systems that can offer rapid mixing of enzyme and substrate and allow for real time measurement of initial rate are essential.

This review summarizes works on enzyme kinetics studies using various formats of flow-based analysis techniques. The trends in down-scaling flow-based systems as micro- and nanodevices are also included. Flow-based systems provide high-throughput study with precision that conventional manual experiments cannot offer.

## 2. Flow-Based Analysis Systems

### 2.1. Evolution of Flow-Based Analysis Systems

Since its invention in the 1970s, flow injection analysis (FIA) technique has gained interest for numerous applications [8, 9]. The system is based on dispersion of sample and reagent solutions in small tubing by flowing and merging them together. Product is formed while the merged solutions are flowing into the detector. Special flow-through-cells are made for continuous flow of solution into and out of the detector and many are commercially available for various detectors [10–15]. This enables flow injection analysis-based systems to couple with various types of detectors (e.g., electrochemical, mass spectrometry, chemiluminescence) [16–19] other than visible absorption spectrophotometers that are usually employed in enzyme kinetic studies.

Common components of the flow system are a pump for delivering continuous flow of a carrier stream, an injection valve for introduction of sample, a mixing coil for promoting the production of product, a detector, and small plastic tubing used to connect all the components. In cases where reagents are rare or expensive and samples are abundant (e.g., environmental samples such as river/sea water), one may inject reagent into a stream of sample solution instead of vice versa to save reagent [20]. This is called reverse FIA system and it may be suitable for study of enzymes since it offers the benefit of low enzyme volume consumption. A simple single line FIA system can be used to conduct a simple chemical reaction using very small volume of solutions, that is, a few tenths to hundredths microliters of injected sample. Detection is done before the steady state is reached but at precise timing by controlling the constant flow rate of solution. First-generation FIA system can accommodate multiple reagents using a T- or Y-shaped connector to merge more reagents into the carrier stream. Multicomponents such as valves, syringes, and pumps can be added to later generation multicommutated FIA systems to allow for better handling and more flexible formats of operation [21, 22].

Various sample pretreatment processes may be coupled with the FIA system. Examples are solvent-solvent extraction [23, 24], sample clean-up such as filter and dialysis [25–31], digestion [32], dilution [33], and preconcentration [34–36]. The technique so called bead injection, in which active microbeads are used as solid surface to accommodate chemical reaction or to selectively trap/separate and accumulate analyte of interest from sample matrices, has provided for more applications [37–39]. Beads with suitable sizes, that is, small enough to be moved with the flow of solution but big enough not to clog the valve and the tubing, have been used as solid phase separation media. These beads can be discarded after each analysis to prevent memory effect.

Sequential injection analysis (SIA) technique is downscaled from the FIA system, using a syringe pump, which is controlled by a computer software, to deliver a small microliter volume of solution. A multiports selection valve is suitable for handling multireagents/samples. Reagents and sample plugs are drawn into the holding coil in stacked zones fashion, before being pushed in reverse direction into the detector [40, 41]. SIA system can accommodate reactions that require precise small volume and time control including enzyme labeled bio/immunoassay [42]. It is also a useful tool for online sample pretreatment [43]. Applications of SIA systems for enzyme-based assay have recently been reviewed [44]. There are numerous reports on applications of SIA involving enzyme usage and enzyme studies. The main reason may be owing to the smaller volume and multireagents handling capabilities of the SIA which make it more suitable for high cost enzymes/reagents and more complicated multisteps reactions. A special unit, made of plastic (available in polyethyl ethyl ketone PEEK and polymethyl methacrylate PMMA) with small and precise microchannel, incorporated onto a normal multiselection valve was introduced into the market as “Lab-on-valve” (LOV) [45]. The LOV also includes an optical fiber detection unit inserted into the selection valve to measure spectrophotometrical changes of the solutions that flow through the flow cell channel which is part of the selection valve. Real-time measurement is possible [46], although it is still limited to spectrophotometric changes only. Lab-at-valve (LAV) is another way to perform analysis similar to LOV, but without the special unit placed on the face of the multiselection valve. Rather, the detection unit is connected to one of the selection valve ports at close proximity to reduce the traveling time prior to detection [47, 48].

With state-of-the-art technologies and advances in material science, lab-on-chip (LOC) has emerged as a new way of analysis. The idea of solution introduction, mixing, and moving of the solution from inlet to detector to the outlet is very similar to that of the FIA/SIA-based techniques, but it usually requires different means to perform the task (e.g., capillary flow, electroactivated pumps, gravity force, acoustic wave, etc.) [49–52]. 

### 2.2. Flow Profile

**Figure 1:**
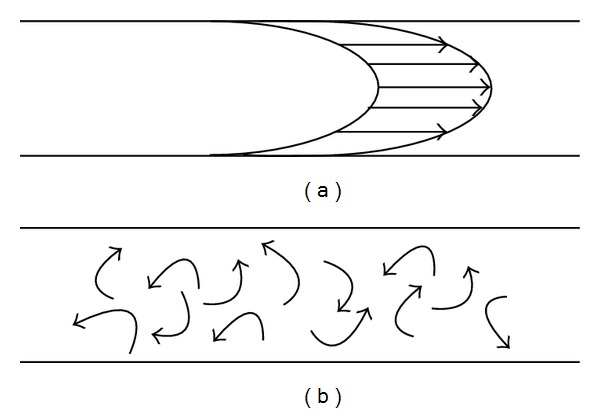
(a) laminar flow and (b) turbulent flow.

The nature of the flow profile of the plug of solution introduced into the flowing stream of carrier solution is normally affected mainly by laminar flow.  Figure 1(a) illustrates the laminar flow effect in a small tubing which distorts the solution plug into parabolic profile in the middle of the zone. The resistance between solution and the wall of the tubing causes the solution to travel slower near the wall of the tubing. In the past, researchers tried to make sure to reduce the laminar flow effect by dominating it with turbulent flow, Figure 1(b), which can be enhanced with the aid of a winding mixing coil to promote a homogeneous product zone and to minimize peak distortion [53]. However, some reports [54–56] demonstrated that laminar flow in the small tubing does not have that much effect on distortion of the zone to the point that it affects the accuracy of kinetic studies. Therefore, a simple continuous flow is possible for kinetic studies [7]. Nevertheless, this may depend on various factors including geometry and design of the flow manifold, and the nature of sample being studied. Therefore, other flow-based formats have been introduced to compromise the effects of diffusion, dilution, laminar flow, and nonsteady-state feature of the flow-based technique.

## 3. Applications of Flow-Based Analysis Systems in Enzyme Kinetics Studies

### 3.1. Flow-Based Systems versus Manual Operation for Kinetic Studies

In a conventional study of enzyme kinetics, manual bench top operation is done by mixing solutions of an enzyme of constant concentration with various concentrations of substrate. Spectrophotometric changes of the reaction mixture are recorded immediately to obtain data at the initial rate. The inconsistency of mixing time before detection and the time wasted of manually placing/moving cuvette in and out of the spectrometer can cause some errors. Most kinetic studies may accept the 5–20% error of this manual operation [7]. However, to be more precise and to gain an additional improvement of analysis time, semi-to-fully automatic flow-based systems may be considered as better alternatives.

Flow-based systems can introduce the product zone into the detector very quickly which enables measurement close to initial state. Although, one may expect that the dispersion and dilution of the product zone in the flow line may affect the accuracy of the results, many reports showed successful kinetic studies of enzyme in continuous flow injection systems [7, 54, 56]. The key to this is to minimize the dilution and dispersion as much as possible by reducing traveling time/traveling distance of the product zone to reach the detector. Various flow-based systems have been reported for enzyme kinetic studies with both solution (non-immobilized) and immobilized enzymes. These works are summarized in Table 1 and are described in more detail in the following sections.

### 3.2. Various Flow-Based Systems Developed for Enzyme Kinetics Studies

Classification of flow strategies is based on the manipulation of the mixing zone after enzyme and substrate solutions have merged. In brief, “continuous flow” involves nonstopped flow from introduction to waste, “stopped flow” involves physically stopping the product zone for a period of time before online detection, “quench flow” involves both physically and chemically stopping the product zone and with offline detection, “merging zones/bypass flow” requires a manifold that can change the flow direction to selectively deliver only a certain part of the product zone to the detector, and “air segmented flow” employs small air segments between each solution bolus to prevent early mixing. Advantages and disadvantages of these different flow strategies are summarized in Table 2.

#### 3.2.1. Continuous Flow

**Figure 2:**
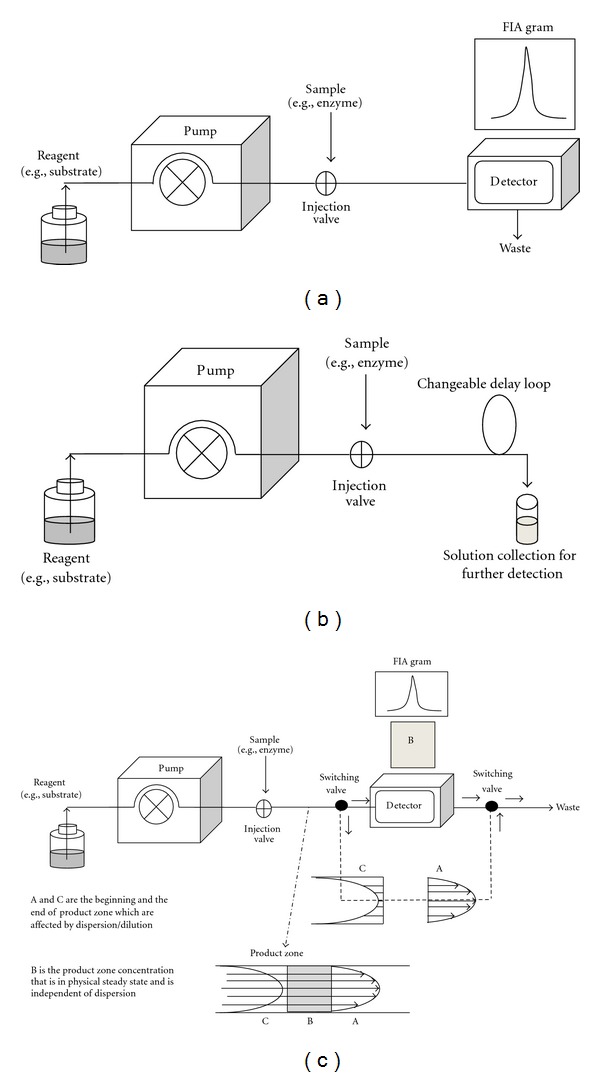
Basic manifold diagrams of (a) continuous and stopped flow, (b) quench flow, and (c) merging zones/bypass flow.

Despite the laminar flow and detection at nonsteady state, these features of the flow injection technique pose no or negligible problems in most cases. A simple continuous flow, Figure 2(a), has generally been applied to automate enzyme kinetic studies. Many applications have coupled the FIA with various types of detectors and sample treatments for different studies evolving enzyme kinetics. Examples are study of enzyme inhibitor [57] including thermal and acid inactivation of enzyme [58, 59], enzyme-substrate mechanisms of single attack [60], effect of immobilization on enzyme activity [61], and effect of magnetic field on enzyme activity [62]. Different arrangements of the manifold can be done such as an open-close flow injection analysis where reagent (i.e., substrate-inhibitor) is trapped in certain part of the manifold and allowed to continuously flow and circulate through the enzyme reactor for kinetic information [63].

One report [7] mentioned an interesting possibility of using high precision system, that is, flow-based analysis, to study kinetic isotope effect. Study of kinetic isotope effect can be used to reveal the reaction mechanism based on the comparison of reaction rates of different isotopically labeled molecules in a chemical reaction. The change of isotope mass dramatically influences the change of the reaction rate. For example, deuterium has mass twice greater than hydrogen causing the rate of reaction involving deuterium bond to be much slower than that of hydrogen bond (e.g., C–D versus C–H bond). The differences in rate are significant and can be measured with manual kinetic study. However, heavier isotopes such as C-12 versus C-13 have only a small mass difference which causes insignificant change in reaction rate that is practically impossible to study with the normal manual technique. 

#### 3.2.2. Stopped Flow

The stopped flow injection technique has been effectively applied for the study of rate of chemical reactions for the past few decades [18, 64–66]. Using the same manifold as a continuous flow system, Figure 2(a), the flow of solution in the tubing can be stopped for a desired period of time by stopping the pump. The reaction time is prolonged and the progress of the stopped reaction zone can be continuously monitored over time. For example, an enzyme-substrate mixture may be stopped in a flow-through cell located in the detector in order to follow the enzyme kinetics and track the changes due to inhibitors. This technique helps to improve sensitivity as compared to continuous flow because measurement takes place when a higher amount of product is being accumulated or when the decrease in reactants is more pronounced. In many industries, enzymes are immobilized on a solid surface for repetitive use. The application of the stopped flow system for study of the kinetics of the immobilized enzyme was reported by stopping the substrate solution at the reactor packed with enzyme coated beads coupled with oncolumn detector, that is, conductometer, for real-time detection [67]. Arrangement of the manifold to reduce operation steps while yielding more kinetic data points was successfully attempted. With the use of double injection valves and split flow lines, enzyme and substrate could be injected simultaneously and merged prior to being split and held for different lengths of stopped time. With this set up, a two-points kinetics study was achieved in one injection [68].

#### 3.2.3. Quench Flow

This technique is based on a similar idea to the stopped flow technique, where enzyme-substrate mixture is stopped within the flow system for monitoring the change in product or substrate concentration over time. The difference is that in the stopped flow technique, one reaction mixture stays in the system (e.g., at the detection cell) for continuous monitoring of change (usually spectrophotometrical change) with time. In quench flow technique, spectrophotometric changes cannot be registered during the reaction. More than one reaction mixture is needed to be introduced into the part of the system called “delay loop or aging loop,” see Figure 2(b). Different reaction times are allowed by changing the size of the delay loop for each replicate, before being stopped by the addition of the “quench solution” [69]. Then, the quenched mixtures of different reaction times are collected. These aliquots are analyzed using separate appropriate methods (e.g., gel electrophoresis and chromatography). This provides the means to isolate, to determine structures of intermediates and products within a reaction, and to gain insight into catalytic mechanisms [70, 71].

Although not many research articles have been published with quench flow analysis for enzyme study, at least two companies offer commercial quench flow systems which are aimed toward use as an alternative system to stopped flow analysis [72, 73]. Rapid flow of solution is desired to ensure turbulent flow for better mixing. Syringe pumps are used for delivering low volume solution. Some models offer a multiselection valve controlled by computer software for selection of preconnected variously sized delay loops that will create various ages of quenched mixtures ranging from a few milliseconds to 100 seconds or more.

#### 3.2.4. Zone Trapping/Bypass Zone Trapping Flow

Zone trapping/merging zones technique or Bypass zone trapping flow injection analysis (ByT-FAS) was introduced in the 1980s with similar principle to stopped flow technique [74–76]. However, it placed more emphasis on ensuring that the part of the sample/reagent plug being measured had reached physical steady-state and had not been affected by laminar flow and dispersion. This is done by controlling the sample size to be sufficiently large and by arranging the flow manifold in such a way that the plug of mixture contains a portion of physical steady state, as shown in Figure 2(c). Only the steady state portion is detected. Other parts of the mixture plug that do not attend the steady state are forced to bypass the detector via a switching valve. Time control in valve switching to change the direction of the flow at the beginning and the end of the product zone to bypass the detector is very critical. The detected zone (the middle of the product zone) is passed into and trapped inside the detector for a desired period of time, similar to the stopped flow strategy. However, in this case, the trapped zone is truly at steady state. Therefore, it is possible to calculate for the more precise concentration of analyte in the detection cell at the time of detection. The bypass zone trapping technique has not been carried out that often, perhaps due to the fact that nonsteady state and laminar flow do not show major error in kinetic study in most cases. By principle, this technique would ensure more accurate results. The latest report on zone trapping technique is the critical evaluation on its performance as compared to the zone stopped flow technique [75], but it was for determination of nitrite, not enzyme. It was found that both techniques offer the same figures of merit, except the sampling frequency is higher with the zone trapping technique.

#### 3.2.5. Air-Segmented Flow

The use of air segments to separate zones of solution and carrier stream, for the purpose of eliminating dispersion/diffusion and prolonging the time prior to mixing, has been reported with sequential injection techniques [77–79]. The syringe pump can control precise volume of air as well as solution. Recently, the sequential injection lab-at valve (LAV) system was developed for rapid measurement that mimics batch manual enzyme kinetic study [80]. To ensure the measurement of enzymatic reaction at initial state, air segments were introduced in between each solution plug to prevent early mixing of the enzyme and substrate zones. Similarly, air segments were also used to separate solution zones at both ends of the stacked solution plugs from the carrier stream to prevent dilution and dispersion. An open end reactor was directly connected to one of the ports of the multiselection valve. When the enzyme and substrate zones along with air segments are introduced into the reactor, air segments will not cause any problem in the spectrophotometric measurement because they will exit to the atmosphere. A fiber-optic unit directly located at the reactor could monitor real-time initial rate of the reaction as each solution plug was introduced and mixed in the reactor.

## 4. Future Trends in Development of Flow-Based Systems for Enzyme Studies

On-site analysis is in demand for many areas of work including industrial quality control, agricultural and environmental studies, food analysis, and health care. All these areas of applications may involve enzyme kinetics and inhibitor studies. Downscaling of the analytical system toward micro-/nanofluidics format has become the trend for the development of future devices. This includes the device application for enzyme kinetics study [81, 82]. Similar to the larger scale flow analysis, microfluidics systems are coupled with various methods to enhance the sensitivity of the measurements. Using porous matrix packed in microchannel as stationary phase for substrate [83], monolith and coating enzymic reactor [84], stopped flow operation on microfluidics design [85], and coupling a microchip to a high sensitivity detector such as ESI-MS [86] and fluorescence microscope [87] are just some examples.

An application in enzyme study within a single cell [88] has also been reported. Research in the area of using a single cell may be gaining more interest as combined information from various single cells may reveal a better understanding of the living systems as compared to information gained from an average cell population. A microdevice should be suitable for this task. Until now, the main challenges of the micro-chip remain in the area of making an effective tiny pump and detector to be placed on the chip and the utilization of forces or methods of solution delivery that do not require external power.

Searching for new enzymes or identifying a particular enzyme through its fingerprint, in which many parallel enzyme assays under various conditions and types of substrate are combined to gain a reactivity profile of a particular enzyme, has become an important trend in developing enzyme systems that are suitable for certain applications [5, 89]. The high-throughput feature of flow-based analysis should be very welcome for this area of study. The complex data obtained from different kinetic studies may be better evaluated with the help of statistics, chemometrics, and computational modeling. These tools can help to save time and offer better prediction and conclusion about performance and characteristics of enzymes [90] as well as quantitative analysis of enzyme [91].

## Figures and Tables

**Table 1:** Summarization of flow-based method for enzyme study.

Flow-based technique	Enzyme	Methodology	Comments	Reference No.
Continuous flow	Tyrosine phosphatase	Use 3 syringe pumps for delivering solutions with constant combined flow rate of 100 *μ*L/min for reaction with kinetic time scale of a minute or more	General application of flow system based on laminar flow, possible to be applied to faster reactions by reducing the diameter of tubing	[7]
	Alkaline phosphatase	Randomly and site directed immobilized his-tag alkaline phosphatase on beads were studied using FIA-chemiluminescence system	Site directed enzyme had *V*_max⁡⁡_ (activity) higher than randomly immobilized enzyme	[19]
	Acetylcholinesterase	Online dialysis of product before mixing with chromogenic reagent	Online pretreatment	[27]
	Acetylcholinesterase and angiotensin-converting enzyme	SI-LOV system with microreservoir and fiber optic/stirrer	Kinetic parameters obtained agree well with literature values	[41]
	Urease	Thermal inactivation, flow colorimetry, and model equation	Can conclude about reversibility and irreversibility of denature and native forms	[58]
	*α*-amylase	Basic FIA system for measuring enzyme activity at different pHs	Very stable at neutral pH 5–8, but loss of activity out of this pH range	[59]
	Glucanase	Fluorescence probe flow injection	Confirm the use of theoretical equation to predict kinetic parameters	[60]
	Alkaline phosphatase	Immobilized enzyme on Sepharose beads and packed in the reactors	Study effect of orthophosphate inhibitor	[61]
	*α*-amylase	Immobilized with glutaraldehyde on polyurethane foam and study activity under magnetic field	Show oscillatory behavior of enzyme reaction	[62]
	Alkaline phosphatase	Open-closed flow injection, theophylline as inhibitor	Km and inhibitor constant were determined	[63]

Stopped flow	Fructose bisphosphatase	Label free, mid IR detection	Alternative detection method	[57]
	Glutathione transferase	Potentiometric detection for mechanism of different isoenzymes	Alternative detection method	[18]
	Elastase	Conventional stopped flow-spectrometric system for slow binding kinetic approach	Conclude that inhibition is 2 step mechanism and gain insight understanding of the effect of heparin on the inhibition mechanism	[65]
	5-enolpyruvoyl shikimate-3-phosphate (EPSP) synthase	Fluorescence measurement at equilibrium	Evaluation of substrate and inhibitor binding	[66]
	Tannase	Immobilized enzyme on glass beads, packed in conductometric flow cell	Check activity of immobilized tannase which is commonly repetitively used in industry	[67]
	Total and prostatic acid phosphatase	Double injection flow analysis	Increase injection concentration 2 fold to compensate dilution	[68]

	Protein kinase	Phosphorylation of peptide substrate, quench with acetic acid	This is the first chemical observation and characterization of phosphoryl transfer at the active site of protein kinase	[69]
Quench flow	3-Deoxy-D-manno-2-octulosonate-8-phosphate synthase	Anion exchange HPLC for detection of radiolabel	Gain conclusion about reaction intermediate	[70]
	5-enolpyruvoyl shikimate-3-phosphate (EPSP) synthase	Radioactively label the enol moiety and then separate and quantitate products with HPLC after acid quench	Observe tetrahedral intermediate	[71]

Zone trapping/Bypass trapped flow	Hexokinase	Coupled to glucose-6-phosphate dehydrogenase, and monitored production of reduced NADPH fluorometrically	Comparable Km and Ki value to the published data obtained from manual techniques	[74]

Air segmented flow	Peroxidase	LAV with microreservoir with air segments	Mimic manual operation and eliminate dilution/dispersion effect	[80]

**Table 2:** Comparison of the main advantages and disadvantages of different flow formats for use in enzyme kinetics studies.

Flow formats	Advantages	Disadvantages
Continuous flow	(i) The most simple system because of no requirement of extra part or flow manipulation	(i) Potential for low accuracy due to dispersion, dilution, and laminar flow effect (though, reports showed there are no significant problems)
	(ii) High sample throughput with the least time consumption on flow operation	(ii) May be lower in sensitivity than other formats, if not enough reaction time due to high flow rate

Stopped flow	(i) Improved sensitivity by increasing reaction time before detection	(i) Excessive stopped time may cause more dilution/dispersion of the reaction zone which will affect accuracy of the measurement
	(ii) Possible to follow the reaction at various increments of stopped time which may give more information about the reaction	(ii) Lower sample throughput because of longer analysis time due to the stopped time prior to detection

Quench flow	Same as stopped flow format	(i) Extra time consumption in collecting aliquots of quenched solutions for further analysis
		(ii) The least automatic due to separated detection step
		(iii) Requires quench solution to stop chemical reaction

Zone merging/bypass flow	(i) Possible to calculate for accurate concentration of the detected product because only the part of the mixing zone that is not effected by dispersion/dilution is detected	(i) Requires higher injected volume to gain adequate size of the product zone
		(ii) More complicated arrangement, requires extra switching valve to change flow direction of the beginning and the end parts of the mixing zone, and needs precise time controlled operation

Air segmented flow	(i) Accurate concentration because there is no dispersion, dilution, and or laminar flow effect on concentration measurement	(i) Requires SIA system to precisely control small volume air segment which may not be possible if use FIA system
	(ii) Ensures measurement of initial rate	
